# Supplementary material for: Potential Role of Circulating PD-L1+ Leukocytes as a Predictor of Response to Anti-PD-(L)1 Therapy in NSCLC Patients
Source: Biomedicines. 2024 Apr 25;12(5):958. doi: 10.3390/biomedicines12050958 (PMC11117542; doi:10.3390/biomedicines12050958)
Supplement: Supplementary file 1 [file biomedicines-12-00958-s001.zip › biomedicines-2928137-supplementary.pdf]

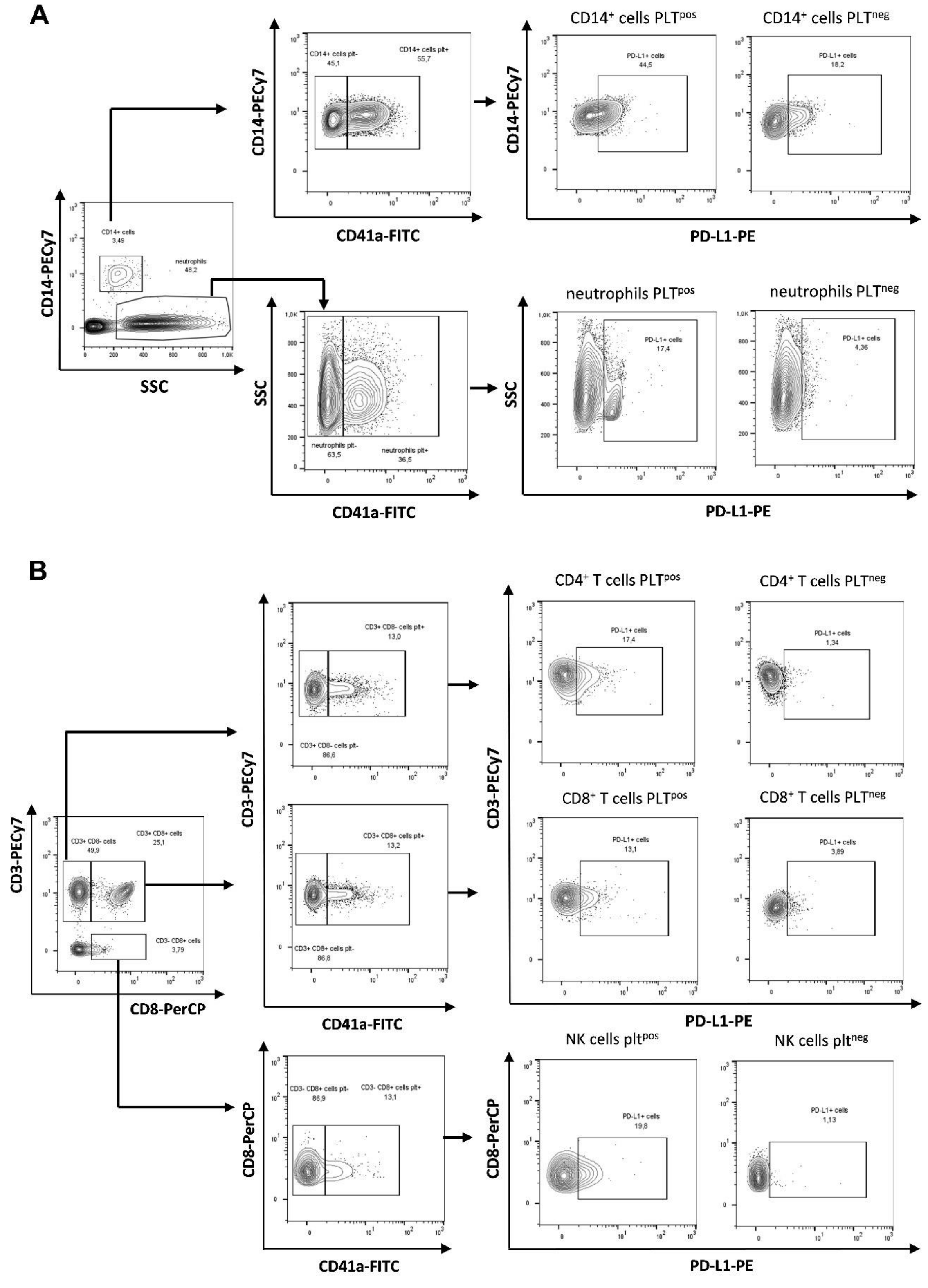

Figure S1. Flow cytometry gating strategy for measuring PD-L1 expression on leukocytes with or without bound platelets. (**A**) Monocytes and neutrophils were initially gated based on CD14 expression and SSC with monocytes being $CD14^+$ cells and neutrophils being $CD14^-$. (**B**) Lymphocytes were initially gated according to FSC-SSC and then classified into $CD4^+$ and $CD8^+$ T cells based on CD3 and CD8 expression. NK cells were identified as $CD3^-$ $CD8^+$ cells. Leukocyte-platelet aggregates were determined by the positivity of the platelet-specific marker CD41a. Expression of PD-L1 was measured in both $CD41a^+$ and $CD41a^{neg}$ cells.

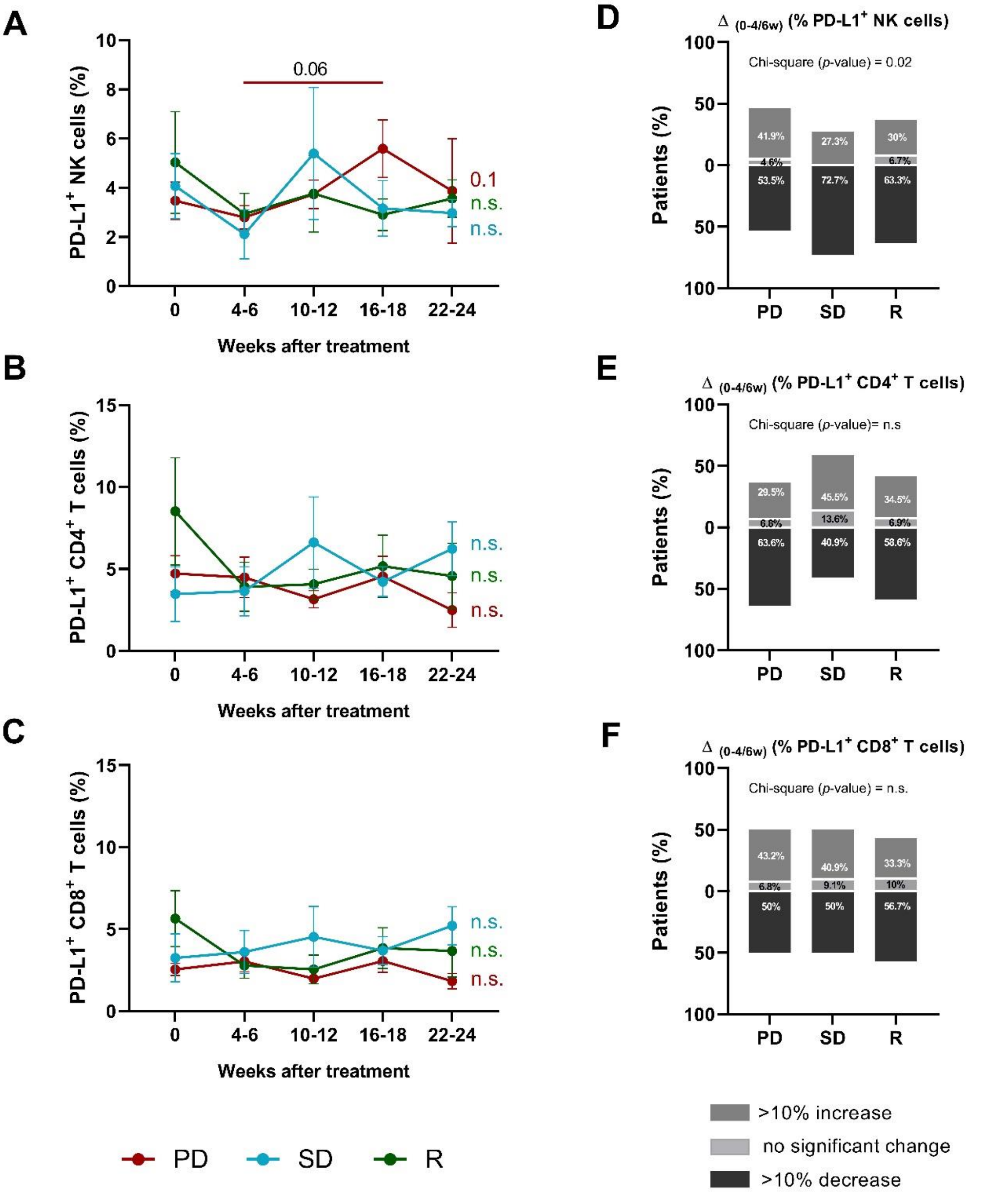

Figure S2. Kinetics of PD-L1+ NK cells and T lymphocytes in NSCLC patients based on anti-PD-(L)1 response. Longitudinal monitoring of PD-L1+ (**A**) NK, (**B**) CD4+ and (**C**) CD8+ T cells percentages during 24 weeks of anti-PD-(L)1 treatment in PD (red line), SD (blue line) and R patients (green line). Changes (>10% decrease (black), no significant change (with less than 10% change) (light grey), and >10% increase (dark grey) in the percentages of PD-L1+ (**D**) NK, (**E**) CD4+ and (**F**) CD8+ T cells in the first 4-6 weeks of treatment. P-values are show in graphs. n.s. is not significant. NSCLC, non-small cell lung cancer; PD, progressors; R, responders; SD, stable disease.

Table S1. Model fitting information of the multinomial logistic regression (obtained by SPSS).

Model Fitting information				
Model	Model Fitting Criteria	Likelihood Ratio Tests		
	-2 Log Likehood	Chi-Square	df	Sig.
Intercept only	94.76			
Final	59.35	35.41	8	0.000

Df, degrees of freedom; Sig. Significance.
